# Life Gets Better: Important Resilience Factors When Growing Up With ADHD

**DOI:** 10.1177/10870547241246645

**Published:** 2024-04-15

**Authors:** Cecilie R. Dangmann, Gunhild K. W. Skogli, Mira E. G. Holthe, Anne K. M. Steffenak, Per N. Andersen

**Affiliations:** 1 Inland Norway University of Applied Sciences, Elverum, Norway; 2 Innlandet Hospital Trust, Brumunddal, Norway

**Keywords:** ADHD, adolescence, resources, resilience

## Abstract

**Objective:**

To explore emerging adults’ descriptions of important resilience factors when growing up with ADHD.

**Method:**

Individual interviews with 10 emerging adults (21–24 years) who participated in a 10-year follow-up study, analyzed using thematic analysis.

**Results:**

The main theme was that “life gets better.” Resilience factors contributing to this positive development were strategies to regulate ADHD, valuable relationships, acceptance, seeing positive attributes of ADHD, receiving tailored, non-stigmatizing support, and participating in meaningful activities.

**Conclusion:**

Growing up with ADHD was associated with both challenges and positives, but the main resilience theme was that life gets better. A variety of resilience factors contributed to this, but relational and environmental factors seemed particularly important. Acceptance, both from society and self-acceptance, were related to all resilience factors in various ways indicating that better knowledge of ADHD might foster better understanding and acceptance of children and adolescents with ADHD.

## Introduction

ADHD is characterized by levels of inattention, impulsivity, and hyperactivity that are developmentally inappropriate, and therefore impact daily activities and social interactions (American Psychiatric Association [APA], 2013). ADHD symptoms are frequently associated with poor academic performance and reduced social functioning (Franke et al., 2018). Evidence also suggests that children and adolescents with ADHD face significant stigmatization (Lebowitz, 2016), bullying (Cuba Bustinza et al., 2022), and an increased risk of co-occurring conditions such as depression and anxiety (Skogli et al., 2013). The overall quality of life (QoL) for children with ADHD is lower than for their neurotypical peers, especially in school and psychosocial domains (Dona et al., 2023). Longitudinal studies show improvements in co-occurring symptomatology and in QoL with age and maturity, but young adults with ADHD still have lower QoL than their neurotypical peers (Orm et al., 2021, 2023).

Growing up with ADHD is therefore associated with significant adversity. Despite this, ADHD is striking in its diversity of developmental outcomes, and negative outcomes are by no means inevitable (Lee et al., 2016). Young people may adapt well despite risk, flourish despite symptoms, or function well in one or more domains even if they struggle in others. Positive adaptation despite risk or adversity, that is, resilience is conceptualized as a dynamic interaction between the individual and context (Masten, 2014). Within the context of child development, socioecological models emphasize how resilience is not just a set of individual capacities or individual adaptation, but also how surroundings adapt—thus resilience models include environmental factors in several dimensions (Ungar et al., 2013). Traditionally, research efforts have focused on risk and negative outcomes associated with ADHD, but strength-based approaches are increasingly highlighted in both research and practice (Grønneberg et al., 2023; Lee et al., 2016; Sedgwick et al., 2019). Studies using a variety of resilience measures report different results, some showing reduced levels of resilience (Regalla et al., 2019) while others show levels similar to neurotypical children (Charabin et al., 2023). In a study comparing children with and without ADHD, both groups were as likely to be described as resilient by their teachers and parents (Chan et al., 2022).

Factors which buffer against negative effects of risk or promote positive outcomes are often referred to as protective or promotive resilience factors (Masten, 2014). A review of resilience factors in youth with ADHD found the strongest evidence for social and family system factors (Dvorsky & Langberg, 2016). Positive parenting and family cohesion promoted wellbeing directly, but also mediated the relationship between ADHD symptom severity and QoL. Family support decreased parental stress, which in turn led to better outcomes for the youth (Barreto-Zarza et al., 2022; Dvorsky & Langberg, 2016). However, social acceptance by both peers and adults seemed to have the greatest impact, buffering ADHD symptom severity, and reducing bullying (Dvorsky & Langberg, 2016). To date, there is less support for individual-level resilience factors, but one longitudinal study from Norway found that better self-esteem and social skills in adolescence, as dimensions of individual resilience, was associated with better psychosocial functioning in early adulthood (Schei et al., 2018). Medication remains the most common treatment for ADHD. However, the value of non-pharmacological interventions (e.g., parenting interventions, Cognitive Behavior Therapy [CBT], and social skills) has been emphasized (National Institute for Health and Care Excellence, 2018). Children who receive treatment, both medication and non-pharmacological, have better QoL, self-esteem, and academic results than children not receiving treatment (Arnold et al., 2020; Dona et al., 2023; Harpin et al., 2016).

As shown, a multitude of factors on individual, developmental, familial, and societal levels contribute to the heterogeneity of adaptive outcomes in ADHD. Conceptualizing resilience in ADHD youth and identifying resilience factors is therefore important to complement our knowledge of risk factors and negative outcomes (Chan et al., 2022; Lee et al., 2016). The voices of the children and youth themselves are important to better understand the process of risk and resilience in growing up with ADHD and their experiences often differ from those of their parents, teachers, or health professionals (Eccleston et al., 2019). When youth describe their experiences of living with ADHD, their stories highlight both positives and challenges (Eccleston et al., 2019). They describe societal stigma and expectations to “fit in”, as well as expressing how they sometimes feel different and misunderstood. At the same time, they describe a maturational shift from being passive to active decision-makers, highlighting the importance of being acknowledged, understood, and taken seriously. Through the process, they developed greater self-knowledge, life skills, and mastery (Chen et al., 2023). With time, they develop several strategies to manage their ADHD, for example educating themselves on the diagnosis, structuring daily tasks, and engaging in activities of personal interest. In addition, social networks, professional support, medication, and adjustments at school or work had positive benefits (Chen et al., 2023). Some express positive self-esteem highlighting their strengths and talents (Eccleston et al., 2019). The following are also viewed as positive attributes of ADHD by individuals themselves: High levels of energy and drive, creativity, thinking “out-of-the-box”, adventurousness, hyper-focus, agreeableness, empathy, and willingness to assist others (Chen et al., 2023; Holthe & Langvik, 2017; Mahdi et al., 2017; Sedgwick et al., 2019). The period of late adolescence and early adulthood is by some labeled “emerging adulthood” (Arnett, 2007) and this might be an important developmental phase to explore considering the maturational shift described in other studies (Chen et al., 2023). Emerging adulthood is characterized by major biological and social changes, including increased self-focus, identity exploration, as well as increasing independence and new demands parallel to the withdrawal of familial and institutional support (Arnett, 2007).

The aim of this study is therefore to explore what emerging adults themselves describe as important resilience factors when growing up with ADHD. Contrary to previous studies, this study did not predefine resilient outcomes (such as academic results or absence of co-occurring diagnoses) to recruit informants but invited the youth themselves to describe their own successes, motivations, and resilience factors.

## Method

This study is part of the Lillehammer Neurodevelopmental Follow-Up Study (LINEUP) of children diagnosed with ADHD, Autism or Tourette’s syndrome. The children were followed over a ten-year period, from referral in late childhood into emerging adulthood, focusing mainly on the development of executive functioning and social/behavioral comorbid disorders measured over three timepoints (*M*age at baseline T1: 12 years, T2: 14 years and T3: 21 years). Participants were recruited from consecutive new referrals to child and adolescent mental health clinics at (*name of county*) Hospital Trust (Norway) and underwent a comprehensive diagnostic assessment at T1 according to national guidelines including clinical assessments, parental and teacher reports, and were given a diagnosis of ADHD according to DSM-IV criteria (APA, 2013). For more detailed information regarding assessment and diagnostic procedures see Skogli et al. (2017) and Orm et al. (2023). The idea for this study originated from several participants expressing an interest in sharing more of their experiences than the quantitative methods allowed. Therefore, a qualitative sub-study was established and 21 of the 65 remaining participants at T3 were invited by letter to participate. These were purposively sampled to achieve a gender balance. Two weeks after the letter, researchers telephoned potential participants and after 10 persons (5 girls and 5 boys) had consented to participate in interviews, no further calls were made. Interviews were conducted about 1 year after the T3 follow-up, using a semi-structured interview guide developed by the researchers and reference group from LINEUP, which included user representatives. The broad topics of the interview concerned experiences of growing up with ADHD and how this affected their lives, relationships and environments in both positive and negative ways. Interviews were done in participants’ own homes or at university premises and lasted between 1.5 and 3 hours. One researcher (MH) conducted the interviews in the fall and winter of 2020 and transcribed the audio-recorded data verbatim, removing all identifiable information. All interviewees received compensation of 500 NOK for their participation and were compensated for travel expenses when relevant. At the time of interviews the participants were between 21 and 24 years (mean age 22 years). Their living situations were diverse; most had completed upper secondary education and were working, most had moved away from home, some lived alone but many shared flats with friends or partners (see Table 1).

**Table 1.** Descriptions of the Participants.

	Gender	Age T1	Diagnose T1 and T2	Age at interview	Diagnose T3	Co-morbidity	Living arrangements
1	Girl	13	ADD	23	Remission	Yes	With partner
2	Boy	14	ADHD	23	ADHD	Yes	With parents
3	Boy	12	ADD	22	Remission	Yes	Alone
4	Boy	10	ADD	22	Remission		With parents
5	Girl	14	ADD	23	Remission	Yes	With parents
6	Girl	14	ADD	24	Remission		With partner
7	Girl	11	ADD	21	Remission		With partner
8	Girl	14	ADHD	23	ADHD		With partner
9	Gutt	12	ADHD	21	Remission		With parents
10	Gutt	11	ADD	21	Remission		With partner
Total	5 girls/5 boys	*M*_age_ = 12.5		*M*_age_ = 22.1			

## Analysis

**Figure 1.**
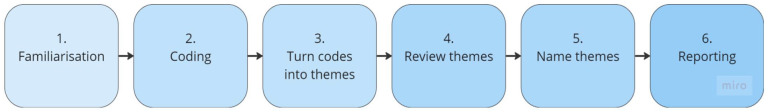
The six-step process in Thematic Analysis based on descriptions by Braun and Clarke (2006).

The interviews were analyzed in accordance with thematic analysis, a method commonly used to identify patterns in meaning (themes) across qualitative data in relation to specific questions. One of this method’s benefits is its flexibility as it can be applied across a range of theoretical and epistemological approaches. In this study, our main interest was the descriptions of resilience and resilience factors, thus a more theoretically driven analysis was adopted, albeit guided by our latent interpretative codes and themes. The analytic process was therefore both inductive and deductive, directed by the six-step process described by Braun and Clarke (2006) (see Figure 1).

Three researchers (CD, GS, and AS) did the preliminary analysis, familiarizing themselves with the data by reading the transcripts and taking notes related to both growing up with ADHD generally and resilience factors specifically. Initially, content was theoretically coded (deductive approach) by coding different descriptions of achievements, stories of mastery, aspirations, and positive factors on three levels (individual, relational, and environmental), guided by socioecological resiliency frameworks (e.g., Ungar et al., 2013 or Masten, 2014). Potential themes were then discussed, and on review, the theoretical codes and themes were found to be broadly overlapping, for example encompassing several levels (e.g., individual and relational). Thus, a more inductive approach was adopted, retracing steps 3 and 4 with all researchers. This resulted in one universal main theme and related resilience factors as themes. Through continued review, sub-themes, and relationships between themes were named and described (steps 4 and 5). At first, the 10 transcripts were equally divided between researchers CD, GS, and AS (steps 1 and 2), but at steps 3 to 5 the different themes were explored by all researcher across all transcripts. Interpretations were discussed at regular meetings with all researchers throughout all steps of the process. An analysis software (f4analyse by Audiotranskription) was used in the initial analysis. To ensure a rigorous, systematic, and reflexive process, we closely followed the methods described by Braun and Clarke (2006), continually guided by their provided checklist of criteria for good thematic analysis (p. 96).

## Results

**Figure 2.**
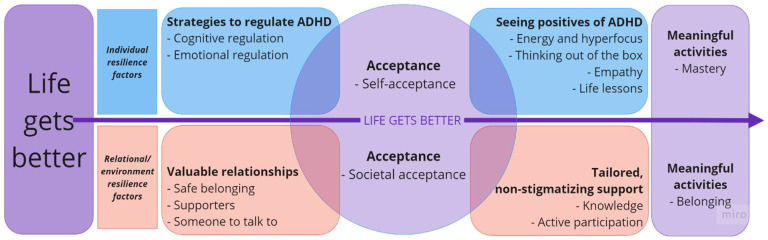
Main theme (Life gets better), themes, and subthemes.

The analysis resulted in one main theme present throughout all interviews—namely that *life gets better* over time. Several resilience factors contributed to this, sorted into six themes. The themes are grouped as factors at the individual level (*Strategies to regulate ADHD* and *seeing positives of ADHD)* or factors related to relationships and the surrounding environment (*Valuable relationships* and *Tailored* and *non-stigmatizing support*). However, two themes bridge both levels (*Acceptance* and *Meaningful activities*; see Figure 2).

### Main Theme: Life Gets Better

Throughout their stories of growing up with ADHD, the participants described both challenges and successes in their lives. Most had experienced difficulties in school and relationships to varying degrees. Some described how side-effects of medication, such as lack of appetite, energy loss, or not feeling like “themselves” had severe impacts on their lives and made them quit medication. Most described experiences of stigma and negative stereotypes related to their ADHD diagnosis, which led to them feeling different or misjudged, and in some cases even being bullied. Some described how they felt that their self-esteem was lowered by teachers and others underestimating their intellect and capabilities.

Despite multiple challenges, most participants felt that they now were accomplished and well-functioning. Some felt their ADHD was worse as a child and now had “weakened.” The main finding is therefore that *life gets better* as participants grow older and mature:

It is quite common, I guess, that as you grow up you grow out of it, at the same time as you find methods to stop it affecting your everyday life. (Participant 3)

The participants also told of multiple and very varied life achievements, such as getting good grades, completing school, getting apprenticeships or jobs, but also being elected to voluntary organizations, moving out, being in relationships, becoming a parent, and helping others. For many, the best period or positive turning point seemed to be during late teens or emerging adulthood—feeling that the things they had dreamt of “were finally happening.” The driving force for many was becoming independent and managing everyday tasks on their own, striving for a not perfect—but “normal” life. The multiple achievements and turning points were linked to several different resilience factors, described below.

### Strategies to Regulate ADHD (Individual Resilience Factor)

The participants wanted to be in control of their ADHD symptoms, essentially to prevent negative effects. They described *cognitive and emotional regulation* strategies, which were often practical, deliberate, and developed over time through trial and error. A frequently mentioned strategy was structuring daily life, for example by using lists, calendars, and reminders. The intention was to avoid forgetting things such as appointments or bills, keep routines for eating and sleeping, to stop worrying about unforeseen things or to feel overwhelmed. Some deliberately avoided relations or situations they felt were chaotic. They wanted system, predictability, and tidiness:

If I tidy around me, it becomes tidy up here (points to head). But if it is messy around me, it also becomes messy up here. (Participant 3)

Many struggled with completing tasks or motivation, and therefore used different strategies to self-motivate. For example, finding the purpose for a particular task, thinking of positive outcomes, reminding themselves of previous experiences of mastery, and positive self-talk. This gave many the determination to try new things, take on challenges, and a drive to push through:

I have thought there is something wrong with me. . .but maybe instead focus on “yes, that is what makes you good at these things”. (Participant 5)

For specific tasks such as exams, tests, or lessons of particular importance, a few mentioned how they used medication just for these situations, or how it helped to have something to fidget with.

Some said they were very sensitive to others’ feelings and easily overreacted emotionally to situations. They had taught themselves to reason with their feelings, to think positively, and be patient. Many also used activities to regulate emotions or distract themselves by being active (going for a walk, to the gym, or dancing) often combined with listening to music or audiobooks. Others favored being social, talking, or creative activities such as making music or drawing.

Self-regulating was energy consuming, and some had learned to let go of control or give into whims to get a break—daydreaming, doing everything at the same time (watching TV, playing games, and being on social media), changing activity when they felt like it, hyper-focusing on something they enjoyed—just accepting the impulses and not feeling guilty. Some had experienced that “last-minute efforts”, although not recommended, usually gave good enough results for them.

### Valuable Relationships (Relational/Environmental Resilience Factor)

Not surprisingly, almost all participants reflected upon the importance of good relationships. These were often long-term, stable, and trusting relationships, and therefore felt like *safe belonging*. When they were with people who knew them well, they could be themselves and not self-regulate or mask, but relax and still feel accepted. For many participants, these close relationships were with family, often parents but also siblings, grandparents, and aunties. Others mentioned long-term friends or partners and a few included professionals such as teachers, school nurses, or doctors.

Another valuable relationship was the role of the *supporters*, described as someone caring, cheering them on, encouraging, and helping when needed:

My grandmother has always been cheering me on. When everybody else was like: “My God, you haven’t accomplished anything”, nothing was ever good enough for my dad. But grandma was always like: “Great! I knew you could do it”. . . A supporter. (Participant 2)

Also important was *someone to talk to* when they needed:

I can talk to both (parents) about these things. And they know. They have been there through everything that has happened. There is no problem talking to them, because they know me very well, and that is good. (Participant 9)

Although talking to someone was important, this was not always straightforward. Participant felt that some ADHD challenges were difficult for neurotypicals to fully understand, and felt it was easier instead to talk to others with ADHD experience. Others were reluctant to talk to their close relations about their challenges, not wanting to worry them. Instead, they talked to their GP, school nurse, or attended support groups but stressed the importance of having good relationships with these individuals for this to be useful.

### Acceptance (Individual and Environmental Resilience Factor)

The stigma of having an ADHD diagnosis was present in most of the interviews. Some felt society saw ADHD as an excuse for ill-mannered kids, laziness, or a sign of bad parenting. Many embodied these negative stereotypes, but also described how they themselves were different from other children with ADHD who were “climbing the walls.” Others had missed having positive role models with ADHD when they grew up, someone “providing hope and inspiration.” Negative stereotypes and expectations to “fit in” led to concealment and secrecy. One recounted their reaction to getting the diagnosis 10 years ago like this:

I wasn’t happy, no. I was mad actually. Like, I said to Mum and Dad: “You’re not allowed to tell anyone”. Not even my sisters were told. (Participant 3)

For some, concealment became a barrier to getting help, especially in school. Many did not want to be treated differently or for teachers to know of their ADHD.

Despite these childhood experiences, the participants were optimistic, believing society was changing for the better. Drawing parallels to increasing openness surrounding mental illness, gender, and sexuality they felt society was becoming more accepting and inclusive:

This is 2020 (. . .) Why wouldn’t they accept us. (Participant 1)

The participants felt that more *societal acceptance* and openness meant the general population now are more knowledgeable about ADHD. Famous people are now open about their ADHD, being positive role models, informing, and shaping the narrative for future generations.

For some, societal acceptance was the key to getting friends and to personally deal with their own ADHD. With maturity came a slow-growing confidence and *self-acceptance*. Learning about ADHD and its symptoms was helpful for some to understand themselves better, and to be open about what they might be struggling with. Confronting stereotypes of ADHD, both societal and their own, was also part of the process of self-acceptance for many. Having gone through an identity process, many felt ADHD was not their only defining feature, but accepted ADHD as a part of who they were:

I feel that accepting symptoms, accepting I am not a broken person or a mistake. . . Instead, I just function in a slightly different way and many others also function in that way. (Participant 6)

Others accepted their own previous transgressions as natural reactions to being undervalued or bullied. Self-acceptance was for some the foundation they needed before being open about difficulties, accepting help, trusting positive feedback, and feeling mastery.

### Seeing Positives of ADHD (Individual Resilience Factor)

In the spirit of acceptance, many managed to also see the positives of having ADHD. Commonly mentioned was having an abundance of physical *energy*, excelling in sports, or having higher work capacity than peers:

Larger energy battery [. . .] that is one of the positive things about ADHD, that you have the energy to just continue. (Participant 10)

Some reflected on being able to use this energy on something positive now that they were older, rather than getting in trouble for it as they did when they were younger. Several participants mentioned *hyper-focus* as a potentially positive factor, being able to work intensively with a topic or skill and becoming good at it:

If I find something I *like* doing, then I am fully focused on that, so I would say that is an advantage. (Participant 8)

Some of the participants mentioned that as their minds worked differently from neurotypicals, they could *think out of the box*, to find new and inventive ways of doing things, to view different perspectives or to be creative:

I like getting new ideas. Maybe take another approach to a problem, to not do things just as they are meant to be done. I think that is an important aspect. And I like being creative, they may be connected. (Participant 5)

Besides seeing the positive attributes of ADHD itself, many reflected on somewhat painful but important *life lessons*; perspectives and skills closely related to the unique experience of growing up with ADHD. Many were proud of their achievements—despite being underestimated, not receiving help or being bullied, they felt that they had survived and learned:

I am one of those who can say “what does not kill you makes you stronger”. I have learned from what I have been through and feel that . . . I now use it to my advantage at work, privately – I try to learn from things I have been through. (Participant 2)

Many felt this had made them more mature, stronger, and better equipped than their neurotypical peers. Some had learned to let go of resentment, look to the future, take life as it comes, or take on challenges. They described how they had worked through difficult periods of their life with determination and perseverance: studying hard to get their diploma, getting an apprenticeship or job, resisting bouts of no motivation, and learning to take pride in their achievements. Some felt adversity had given them more insight, *empathy*, and acceptance of others, developing a stronger sense of justice, describing situations where they had spoken up for others.

### Tailored, Non-Stigmatizing Support (Relational/Environmental Resilience Factor)

The participants provided many examples of practical support they had received within the health services, schools, and at home. Related to descriptions of stigma and acceptance, it became clear that any support needed to be perceived as non-stigmatizing to be useful, but also tailored to each person and context. Two elements seemed to be of key importance for this: *ADHD knowledge* and *active participation.*

Many had experienced that access to support in school depended on receiving a formal ADHD diagnosis, which in turn was dependent on teachers recognizing their difficulties as symptoms of ADHD. Recognizing symptoms of ADHD required *ADHD knowledge*—without knowledge, difficulties were misinterpreted as antagonism, laziness, or stupidity. Many families felt they had to fight to receive support, and in some cases the teacher and their knowledge of ADHD seemed to be the linchpin to access support. If this “linchpin teacher” disappeared—help was often discontinued:

They knew that we were struggling, but they didn’t have enough knowledge about it, so it was just put aside [. . .] Teachers today should be taught more about ADHD, dyslexia and ADD and things like that. What often happens is that they don’t have enough knowledge and then they don’t know how to help. (Participant 1)

According to the participants, ADHD knowledge varied among teachers; newly educated teachers or those with personal experience of ADHD had better knowledge. ADHD knowledge was not only important for recognizing symptoms, but also for being understood. Being understood meant being accepted, respected, and valued, often illustrated by teachers and parents being patient. Participants described patience as “really trying to understand” what support they needed, giving them time and space to succeed—instead of being strict, angry, or impatient. A patient adult did not “take the bait” when students opposed, but instead helped to regulate emotions, giving opportunities to get out of escalating situations. Gentle reminders, such as saying their name if they lost attention in class, worked fine when done with understanding, not as a reprimand. In turn, feeling understood made them feel safe and made it easier for them to ask for help, for example admitting that they did not understand the task given and expressing their need for further explanation. With maturity, some reflected on bad choices they made as young, such as not accepting help or lashing out, realizing this was because they had felt vulnerable and insecure.

Despite describing similar support interventions there was little consensus among our participants as to what was most useful. Some had help from medication, others felt this was only a necessary evil and many had stopped taking it regularly. Many had received help with organizing everyday tasks, reading and writing (e.g., reminders, lists, computers, and sound files of books). Several participants said smaller groups or extra time one-on-one with the teacher made it easier to concentrate, but others did not. Tailoring the support to the individual and context by *active participation* was therefore important:

Listen to the student. Just listen to what he/she, the student, might need help with. If the student does not want help, don’t give any, if he just wants extra time [. . .] just try to help with that. The most important things are communication and the effect. (Participant 10)

### Meaningful Activities (Individual and Relational Resilience Factor)

Many participants described engaging in meaningful activities as something that enhances joy, meaning, and their general quality of life. The starting point was often a special interest in a topic or activity, developing over the years into a hobby, studies, voluntary work, education, or work. Activities such as gaming, sports, and art, but also advocating important issues through organizations was associated with positive feelings, including *mastery*:

I think it is important to find out what makes you flourish, cultivate it and maybe that will help you with the things you struggle with. (Participant 5)

Contrasting the struggles of everyday life, these were positive stories about finding something you were good at, a niche or a skill. Many gave examples of more inclusive environments where being different and having a special interest was actually an asset, not a disadvantage. The experience of mastery was seen as transferrable to other situations such as work. Many were entrusted with added responsibilities, adding to the feeling of contributing to and being a successful member of society.

Special interests or activities were sometimes a gateway to friendship and *belonging*:

The environment is very open for everyone. It’s very inclusive. It’s fun. (Participant 7)

I think it is great to be in “best trick”-competition, for example, doing a trick, landing, it makes you happy. [. . .] (People) want that feeling and they want to do it with friends. (Participant 4)

Meaningful activities offered inclusion into a social community, with people who shared the same interest or passion, and appreciated their competence through positive feedback.

### Summary of Themes

Despite describing many challenges of growing up with ADHD, the main theme in this study was that *life gets better* with age. One individual resilience factor frequently mentioned was gradually finding practical *strategies to regulate ADHD* symptoms, thus coping better with challenges. An important relational factor was experiencing the benefits of close and supportive *valuable relationships. Acceptance*, especially from society, was paramount for the participants as many challenges were related to society’s reaction to their symptoms. Stigma and negative stereotypes were present in all stories, and acceptance from society made it possible for them to accept themselves and to gradually be able to *see the positives of ADHD*. Acceptance was also related to society in general, and school specifically getting more knowledgeable about ADHD, which in turn resulted in receiving better and *tailored, non-stigmatizing support*. Lastly, purposefully prioritizing *meaningful activities* in work and leisure provided enjoyment, mastery, and belonging.

## Discussion

The aim of this study was to explore what emerging adults describe as important resilience factors when growing up with ADHD. The main finding was that despite multiple challenges, most participants now felt well-adjusted and proud of their accomplishments. One participant stressed the importance of inspiring hope by telling others who struggle with ADHD that life *does* get better, expressing the same intent as researchers promoting strength-based descriptions of ADHD (Climie & Mastoras, 2015; Grønneberg et al., 2023). Improvement with age is documented in multiple studies as partial remission, especially symptoms of hyperactivity and impulsivity tend to decline as youth with ADHD age, and fewer symptoms are related to better QoL (Lorenzo et al., 2021; Orm et al., 2023; Skogli et al., 2022). Most of our participants were registered as in “remission” at their last assessment, possibly mirroring the improvements they themselves described. However, longitudinal studies challenge the notion that half the children with ADHD “outgrow” the disorder by adulthood—instead patterns of remission are variable: Many experience a remission of symptoms at some point, but then a recurrence of ADHD after the initial remission. Only 10% show persistent symptoms or persistent remission of symptoms at all checkpoints (Sibley et al., 2022). It may be argued that increased age and maturity in themselves explain the positive development described by our participants, but this contradicts general population studies showing a near-universal *decrease* in life satisfaction between the ages of 10–24 (Orben et al., 2022). It is also important to note that despite improvements in general life satisfaction, youth with ADHD still have lower QoL than their peers (Orm et al., 2023).

Our participants described a variety of resilience factors, from individual to relational as well as environmental factors, contributing to our understanding of adaptative variations in ADHD. Although separated as themes in this study, these factors are obviously connected in multiple reciprocal and complex processes. Many factors resemble well-known general resilience factors such as stable relationships and meaningful activities (Masten, 2014) but described in the context of growing up with ADHD. The purpose of this research was to complement previous descriptions of risk and negative outcomes by purposely looking for positive constructs and resilience factors in experiences of growing up with ADHD. However, nothing is purely positive or negative, but dependent on context as we have attempted to describe. For example, to be open about difficulties may be beneficial in some contexts but not another, and participating in meaningful activities may be a positive supplement to daily life, but not if it takes precedence above all else. This balancing act is also described in other studies of growing up with ADHD (Chen et al., 2023; Grønneberg et al., 2023).

Reconfirming earlier research on resilience factors in ADHD, there was no apparent universal key factor to resilience, but *acceptance* seemed connected to all themes as a positive opposite to stigma and undesirable stereotypes (Dvorsky & Langberg, 2016). The “minority stress model” might therefore be relevant to understand risk and resilience in ADHD youth. The model is previously used to explain health disparities in other minority groups, but increasingly adapted to the minority disability movement and neurodiversity (Botha & Frost, 2020). In this model, experiences of everyday discrimination, internalized stigma and concealment create additional strain (Frost & Meyer, 2023). The view is that the restrictive notions of “normal” create further disadvantages, exposing these minority groups to more stressful life situations with potentially fewer possibilities to cope, thus affecting overall health and QoL rather than specific symptoms. Feelings of otherness, trying to fit with societal expectations but failing, or feeling misunderstood are shown in multiple studies of ADHD (Chen et al., 2023; Eccleston et al., 2019; McMenemy & Nicholas, 2022). The continual effort of being “normal” is related to concepts of masking or social camouflaging where individuals hide their ADHD to fit in, be liked, or avoid negative reactions (Ginapp et al., 2023). Masking as a response to social stigma is more widely described in people with autism and related to delays in diagnosis, internalizing symptoms, and burnout (Pearson & Rose, 2021). ADHD masking is described as concealing your diagnosis, being invisible, overcompensating, or causing disruptions to avoid attention to problems (Ginapp et al., 2023), behaviors that are all present in our participants’ stories. Acceptance therefore seems to be a central resilience theme, essential for reducing some of the additional experienced burden described in the “minority stress model” (Frost & Meyer, 2023). Related to this, our participants described how important safe and accepting relationships were, because they could just relax and be themselves with these people. They also described how they had learned to differentiate when it was not necessary to use strategies to regulate their ADHD, but just let go of control and relax. With age, many also became selective in choosing studies, job, or activities with more accepting environments with wider perceptions of “normal”, where they could be themselves and their skills were appreciated. Feeling different and striving to seem normal is also described in much older adults (Nyström et al., 2020), thus not necessarily dependent on age but societal acceptance in itself. Acceptance, closely connected with ADHD knowledge, could therefore be important themes to explore in further research, possibly using perspectives from the minority stress model.

Coping or thriving despite challenges is the very definition of resilience (Masten, 2014). The varied descriptions of achievements from our participants, contribute to our understanding of resilient outcomes beyond those often chosen as objective study parameters (e.g., school grades or absence of mental distress) and illustrates how resilient outcomes can co-exist with the presence of negative factors such as symptoms and stress (Lee et al., 2016). Growing up with ADHD has been described as a continuous, non-linear process of continually tapping into strengths to tackle ADHD challenges, toward being the best version of themselves (McMenemy & Nicholas, 2022). This closely resembles this study, where the youth desired control over symptoms, independence, and a “normal life.” Similar to the concept of perseverance, this is a continuous and dynamic effort demanding determination and grit, as challenges change over time, environments, and maturity levels (McMenemy & Nicholas, 2022). As such, this description of resilience differs from those where persons return to an original state after considerable trauma and are “healed” (recovery; Masten, 2014). Instead, the youth continuously spent energy in the process of maintaining “a normal life”, as described above. In addition, they described the subtheme of life lessons; experiences of hardship from growing up with ADHD which had made them stronger and more mature than their peers. This resembles descriptions of resilience more as a “steeling effect”, where engagement with stress serves to prepare the individual for better subsequent adaptation (Masten, 2014). The differences in types of resilience may be related to the types of challenge, in this case a steeling effect created by growing up with a long-term disadvantage in a neurotypical normative society, as opposed to recovery after a short-term traumatic experience.

The hopeful message of life with ADHD getting better with time is important, but resilience must not be an excuse for not providing support. Although our participants felt that life got better with age, many experience a recurrence of symptoms depending on age and context (Sibley et al., 2022). In a study where parents and professionals were asked to define resilience in ADHD, they were hesitant to use the word as they felt it was misused to minimize challenges or to pass the responsibility for problems onto the child rather than the school (McMenemy & Nicholas, 2022). In general, treatment, intervention, and support are associated with better outcomes in ADHD children (Dona et al., 2023). Many of our participants had struggled to access help when they needed it or experienced discontinued support, and both seemed dependent on key personnel having adequate knowledge of ADHD. Although getting a diagnosis was a negative experience for some, it seemed important as an “official” recognition for needing support. A study of women who were only diagnosed as adults describe how getting a diagnosis was also important for self-acceptance, after struggling and blaming themselves for their shortcomings for years (Attoe & Climie, 2023). We also found that it was not necessarily the *type* of support that was important, but *how* it was provided; the help should be non-stigmatizing and tailored to the person and situation. Again, the key to this seemed to be ADHD knowledge or experience. In a review of 29 interventions for ADHD teacher training, the interventions significantly increased teacher’s knowledge about ADHD, but did not necessarily change their behavior (Ward et al., 2022). A small selection of these teacher training interventions also managed to change teachers’ negative attitudes toward ADHD, with researchers suggesting that ADHD knowledge made the teacher more tolerant and patient with ADHD students (Shehata et al., 2019).

## Limitations

The participants in this study were purposefully selected to provide a gender balance and compensated, and may not represent the true variety of experiences of growing up with ADHD. Considering the substantial influence of contextual factors, growing up in Norway may be significantly different from other countries. Despite this, the main results seem to reflect previous research in the field. The perspective in this study was emerging adults’ own experiences of childhood and youth, which may create a recall bias. Situations and actions of others are also interpreted through their eyes and do not represent actual intent or facts. Men and women were equally represented in this study, but previous research has shown that they often have different experiences (Attoe & Climie, 2023), something which may be interesting to explore further. All our participants received their ADHD diagnosis as children and considering that the diagnosis was related to self-acceptance and access to support, persons receiving the diagnosis later in life may have other experiences than our participants. After reassessment in this study, several of our participants no longer met the criteria for an adult ADHD diagnosis. However, all participants had received an initial diagnosis which was reconfirmed at T2. As the main purpose of the study was to reflect upon *growing up* with ADHD, not just current situations, their narratives are still relevant. Including only adults with current ADHD diagnoses may have led to different results, as their ADHD may have more influence on their daily functioning.

As researchers, we construct our results through the way in which we interpret the data material at hand. We are aware of our preconceived notions affecting both the interview process and interpretation of results (Braun & Clarke, 2006). To avoid unnecessary bias, the researchers have continually discussed potential interpretations from several fields of research and practice (psychology, public health, and education). Other positionalities, such as socioeconomic status or gender were not considered to the same extent, which might be a limitation. The initial focus on theoretical thematic analysis (Braun & Clarke, 2006) within the fields of resilience research may have aided the comparison to other resilience studies but led to potential loss of more data-driven or latent themes, despite the authors retracing steps 3 and 4 with an inductive approach. At the same time, using both approaches may have created richer descriptions of themes.

## Implications for Interventions/Practice

Based on the current research and the main theme of our analysis, “*life gets better*,” the apparent implication for practice is the commitment to convey hope to children with ADHD and their parents. When children/adolescents and their parents are referred for assessment and diagnosis they often have years of significant challenges behind them such as feelings of otherness, failing to fit societal expectations, feelings of guilt, conflicts, and worries. This may give way to stress and experience of stigma and shame. Neurodevelopmental conditions are often associated with persistent challenges and “poor prognoses.” For some children and parents, the diagnosis can be perceived as a gloomy forewarning of what lies ahead. Yet, given the present body of research, the health care professionals can say that most likely life will get better with time. Contrary to the idea that the ADHD symptoms will remain equally troublesome throughout the span of life, health care professionals can explain how the impact of the symptoms can vary according to the life conditions and context of the child and family.

Furthermore, much can be done to enhance the potential beneficial effects of the resilience factors described in this article. However, this will require a systemic way of working with ADHD. Traditionally interventions have focused on the individual aiming to reduce symptoms and improve coping skills of the specific child and its family, such as medication and practicing regulating strategies. Valuable and effective as these interventions may be in strengthening individual resilience factors, our findings suggest that a broader and more contextual perspective on interventions would be useful. The construct of resilience reflected in our study is aligned with resilience understood as multiple reciprocal and complex processes, where the characteristics of the individual and the social systems interact. The implications for clinical practice are a need for an enhanced focus on the possibilities to build resilience around the child and not just *in* the child.

The current study suggests that acceptance is connected to all themes of resilience as a positive opposite to stigma, contributing to “life getting better” for the emerging adults. Acceptance is a relational process where self-acceptance is intertwined with acceptance from other people and from society. The minority stress model could be a useful theoretical foundation when providing guidance, along with intersectionality and affirmative psychology (Frost & Meyer, 2023). From the perspective of the emerging adults, acceptance from others was associated with the level of ADHD knowledge. Teaching programs on ADHD for professionals working with children and their families such as teachers, health care, or social workers, may therefore be a useful way of building resilience around these children. This can improve timely referral for diagnosis and support, reduce stigma, and improve understanding of their struggles, thereby making tailored support more accessible.

Lastly, active participation in meaningful activities seems to be a central theme connected to the overall resilience process. Both the sense of belonging and the experience of mastery are important aspects of this participation. Interventions focusing on making meaningful activities available for the child (and family) could have multiple benefits. To achieve this, a cooperation between health care professionals and the local environment of the child and family are called for.

## Conclusion

Growing up with ADHD was associated with both challenges and positives, but the main theme in this study was that life gets better. Important resilience factors were strategies to regulate ADHD, valuable relationships, acceptance, seeing positives of ADHD, tailored and non-stigmatizing support, and meaningful activities. Societal negative reactions to diagnosis and challenges related to ADHD seemed to be important influences, and the minority stress model might help explain some of these experiences and reactions. Improved ADHD knowledge and awareness may be important for better understanding and acceptance.
